# Impact of fish consumption on all-cause mortality in older people with and without dementia: a community-based cohort study

**DOI:** 10.1007/s00394-022-02887-y

**Published:** 2022-06-24

**Authors:** Aishat T. Bakre, Anthony Chen, Xuguang Tao, Jian Hou, Yuyou Yao, Alain Nevill, James J. Tang, Sabine Rohrmann, Jindong Ni, Zhi Hu, John Copeland, Ruoling Chen

**Affiliations:** 1 Faculty of Education, Health and Wellbeing, University of Wolverhampton, Wolverhampton, WV1 1DT UK; 2 Institute of Epidemiology and Health Care, University College London, London, UK; 3 School of Medicine, John Hopkins University, Baltimore, USA; 4 College of Public Health, Zhengzhou University, Zhengzhou, Henan China; 5 School of Public Health, Anhui Medical University, Hefei, China; 6 Epidemiology, Biostatistics and Prevention Institute (EBPI), University of Zurich, Zurich, Switzerland; 7 School of Public Health, Guangdong Medical University, Zhanjiang, China; 8 School of Health Administration, Anhui Medical University, Hefei, China; 9 Institute of Psychology, Health and Society, University of Liverpool, Liverpool, UK

**Keywords:** Dementia, Fish consumption, Mortality, Older people

## Abstract

**Background:**

Increased fish consumption reduces the risk of dementia. However, it is unknown whether fish consumption reduced all-cause mortality in people with dementia. The purpose of the study is to investigate the association of fish consumption with all-cause mortality in older people with dementia versus those without dementia.

**Methods:**

Using a standard method of the Geriatric Mental State, we interviewed 4165 participants aged ≥ 60 years who were randomly recruited from five provinces in China during 2007–2009 to collect the baseline data of socio-demography, disease risk factors, histories of disease, and details of dietary intakes, and diagnosed dementia (*n* = 406). They were followed up for vital status until 2012.

**Results:**

The cohort follow-up documented 329 deaths; 61 were in participants with dementia (55.3 per 1000 person-years) and 224 were those without dementia (22.3). In all participants, the risk of all-cause mortality was reduced with fish intake at “ ≥ twice a week” (multivariate-adjusted hazard ratio 0.58, 95% CI 0.34–0.96) and at “once a week or less” (0.79, 0.53–1.18) compared to “never eat” over the past two years. In participants without baseline dementia, the corresponding HRs for all-cause mortality were 0.57 (0.33–0.98) and 0.85 (0.55–1.31), while in participants with dementia were 1.36 (0.28–6.60) and 1.05 (0.30–3.66), respectively.

**Conclusion:**

This study reveals that consumption of fish in older age reduced all-cause mortality in older people without dementia, but not in people with dementia. Fish intake should be increased in older people in general, prior to the development of dementia in the hope of preventing dementia and prolonging life.

## Introduction

Consumption of fish reduces incidence of cardiovascular disease (CVD) [1], respiratory diseases [2], cancers [3], diabetes [4, 5], and mental illness [6]. Our recent study also demonstrated that increased consumption of fish was associated with a reduced risk of dementia [7]. However, it is unknown whether fish intake reduced mortality in people with dementia, and improved survival in older population. Previous studies showed that increased consumption of fish reduced all-cause mortality [8, 9]. Almost all previous studies [8] were carried out in young and middle-aged populations. A few studies have examined the impact of fish intake in older age on all-cause mortality, while the dietary patterns between young-middle and older population are different (e.g., older people had reduced fish consumption [10, 11]). Furthermore, most studies examining the impact of fish intake on mortality were undertaken in high-income countries (HICs), and the findings may not be generalisable to those in low- and middle-income countries (LMICs), where socioeconomic deprivation, cardiovascular disease and risk factors (CVDRFs), social support, and health inequalities are different from those in HICs [12]. China is the largest LMIC with population ageing and has 241 million older people. There have been 15 million Chinese living with dementia [13, 14]. In this study, we examined the data of a community-based cohort study of older people living in China, to assess the impact of fish intake in older age on all-cause mortality in older people with and without dementia.

## Methods

### Study populations and baseline survey

The study population was derived from the Anhui province cohort (the third-wave health survey) and the four provinces' health survey study in China [15]. Their methods of the baseline survey and follow-up have been fully described in previous publications [15, 16].

Briefly, in the Anhui cohort study, we recruited a random sample of 1810 older people aged ≥ 65 years who had lived for at least 5 years in Yiming subdistrict of Hefei city in 2001, and 1709 older people aged ≥ 60 years from all 16 villages in Tangdian District of Yingshang County in 2003. Our trained survey team from the Anhui Medical University used the standard methods of the Geriatric Mental State (GMS) questionnaire [17] and a general health and risk factors record [12] to interview 3336 participants (1736 from the urban sample in 2001 and 1600 from the rural in 2003) for baseline data collection (wave 1). After completing the wave 2 interview involving 2608 participants in the year 2002 for the urban participants and in 2004 for the rural participant [18, 19], we carried out the third-wave survey during 2007–2009 and successfully re-interviewed 1757 participants, obtaining a response rate of 82.4% of surviving cohort members [15, 19]. In the wave 3 survey, apart from the GMS and the general health and risk factors record which was derived partly from the Minimum Data Set (MDS) of the Medical Research Council Ageing in Liverpool Project-Health Aspects (MRC-ALPHA) study [20, 21] and the Scottish MONICA surveys [22], we included other components of the 10/66 algorithm dementia research package [23] and dietary intake questionnaire [7, 15] for interview. Permission for interview and informed consent were obtained from each participant or, if that was not possible, from the closest responsible adult. We recorded details relating to socio-demography, lifestyles, social networks and support, CVDRFs, dietary intakes, and histories of chronic diseases for each participant. All participants were asked to state their dietary intake frequencies which included meat, fish, egg, fresh vegetables, fruits, over the past 2 years in a choice of (1) Never eat, (2) Once a week or less, (3) > Once a week and < daily, (4) Once a day, and (5) > Daily in a simple food frequency questionnaire [7]. According to standard procedures [22], we measured systolic and diastolic blood pressure, height, weight, and waist circumference in each participant. We performed a computer program-assisted diagnosis, the Automated Geriatric Examination for Computer Assisted Taxonomy (AGECAT) [17], to assess the information from the GMS to identify the principal mental disorders and diagnose depression and dementia in the participants [12, 18, 24].

The methods employed in the four-province study have been fully described before [16, 24]. In brief, in 2008–2009, following our wave 3 survey of the Anhui cohort study [7, 15], we chose one urban and one rural community from each of four provinces (Guangdong, Heilongjiang, Shanghai, and Shanxi) as the study fields and sought to recruit 500 or more participants from each community. We employed a cluster random sampling method to select residential communities from each of the four provinces. The target population consisted of residents aged ≥ 60 years who had lived in the area for at least 5 years. Based on the residency lists of the district and village committees, we recruited a total of 4314 participants, with an overall response rate of 93.8%. The protocol of the interview was the same as that in the Anhui cohort wave 3 survey described above.

### Follow-up of the multi-province cohort

We took 6071 participants (4314 from the four-province study and 1757 from the Anhui wave 3 survey) as baseline cohort members, since their interview included the dietary intake questionnaire. In 2010–2012, we followed up the cohort to monitor their vital status and re-interviewed surviving participants using the same questionnaires as those at baseline [16, 19, 25]. The interview team of each province visited the local residential areas to obtain the survival status of each of the cohort members through the resident committees, village/district leaders, and local police stations. There were 329 deaths documented in the cohort. A standard verbal autopsy questionnaire was employed to further identify causes of death from family members, relatives, neighbours, or friends of the deceased. We successfully re-interviewed 3836 surviving participants [16, 19, 25]. The overall follow-up rate of the cohort was 68.6%. Ethical approval for the study was obtained from the Research Ethics Committee, Anhui Medical University, China, and the Research Ethics Committee, University of Wolverhampton, UK (Ref. A1- Favourable, granted in 2010).

### Data analysis

Descriptive statistics were used to examine the characteristics of the participants. Distributions of sociodemographic and risk factors between surviving and deceased were assessed by a t-test for continuous variables and a Chi-square test for category variables. Cox proportional hazards regression models were employed to assess all-cause mortality in relation to consumption of fish over the past two years at baseline. According to this cohort data, we divided the participants into three groups based on their consumption of fish at baseline; (1) “Never eat”, (2) “A little” (including those of “Once a week or less”), and (3) “A lot” (including those of “ > Once a week and < daily”, “Daily” and “ > Daily”). We computed the hazard ratios (HR) and 95% confidence intervals of all-cause mortality at each group level of fish consumption. In the models, we adjusted for different sets of confounding co-variables, including age, sex, province, urban–rural living, educational level, occupational class, income, smoking status, alcohol consumption, body mass index (BMI), marital status, frequency of visiting children or other relatives, activity of daily living (ADL), hypertension, heart disease, diabetes, depression,  dementia, and consumption of meat, vegetables, and fruits. Following the data analysis for all participants, we stratified data of participants with and without dementia at baseline for analysis and tested the differences in HRs between two groups of participants according to those we did in previous papers [19].

All data analyses were conducted using SPSS version 26 software (IBM Co., Armonk, NY, USA).

## Results

Of 4165 participants, the mean age (SD) was 72.1 (7.3) years, 55.3% were women, 58.5% lived in rural areas, and 47.6% were illiterate. The details of the baseline characteristics of the study participants are shown in Table 1. Compared to those surviving, participants who died were older, male, smoking, underweight, less educated, widowed and living alone, and had unsatisfactory income, higher levels of children/other relatives visiting > once a week, higher ADL (i.e., more dependent), hypertension, and dementia. They consumed less fish over the past two years (Table 1). Other factors in Table 1 showed no significant differences between deaths and survivals.

**Table 1:** Distribution of sociodemographic and clinical characteristics of participants: five province study, China

Variable	All		Death		Alive		p*
	Participants *N* = 4165		*n* = 329	(%)	*n* = 3836	(%)	
Age (years)							
Mean (SD)	72.1	7.32	76.7	7.64	71.7	7.16	< *0.001*
Sex (*n*, %)							
Women	2304	55.3	150	45.6	2154	56.2	< *0.001*
Men	1861	44.7	179	54.4	1682	43.8	
Urban–rural living							
Urban	1730	41.5	135	41.0	1595	41.6	*0.847*
Rural	2435	58.5	194	59.0	2241	58.4	
Province							
Anhui	1014	24.3	70	21.3	944	24.6	
Guangdong	902	21.7	74	22.5	828	21.6	*0.340*
Shanghai	926	22.2	71	21.6	855	22.3	
Heilongjiang	460	11.0	33	10.0	427	11.1	
Shanxi	863	20.7	81	24.6	782	20.4	
Smoking status							
Never-smoking	2576	61.8	182	55.3	2394	62.4	*0.010*
Current- or Ex-smoking	1537	36.9	143	43.5	1394	36.3	
Unknown	52	1.2	4	1.2	48	1.3	
Alcohol drinking in the past two years							
Never	3045	73.1	228	69.3	2817	73.4	*0.130*
Current- or Ex-drinking	1051	25.2	94	28.6	957	24.9	
Unknown	69	1.7	7	2.1	62	1.6	
*BMI (kg/m*^*2*^*)*^*†*^							
Cut-off point							
< 20	816	19.6	97	29.5	719	18.7	< *0.001*
20– < 23	1428	34.3	112	34.0	1316	34.3	
23– < 26	1063	25.5	62	18.8	1001	26.1	
> = 26	651	15.6	37	11.2	614	16.0	
Unknown	207	5.0	21	6.4	186	4.8	
*Socioeconomic status*							
Educational level							
Illiterate	1984	47.6	198	60.2	1786	46.6	< *0.001*
Primary school	1100	26.4	69	21.0	1031	26.9	
Secondary school	548	13.2	27	8.2	521	13.6	
> = High Secondary school	325	7.8	23	7.0	302	7.9	
College/University	175	4.2	10	3.0	165	4.3	
Unknown	33	0.8	2	0.6	31	0.8	
Main occupation							
Peasant	2321	55.7	195	59.3	2126	55.4	*0.384*
Manual labourer	628	15.1	42	12.8	586	15.3	
Official/Teacher	536	12.9	39	11.9	497	13.0	
Business	32	0.8	1	0.3	31	0.8	
Housewife	338	8.1	32	9.7	306	8.0	
Others	278	6.7	18	5.5	260	6.8	
Unknown	32	0.8	2	0.6	30	0.8	
Annual income^‡^							
Very satisfactory	333	8.0	23	7.0	310	8.1	*0.013*
Satisfactory	1828	43.9	124	37.7	1704	44.4	
Average	1653	39.7	142	43.2	1511	39.4	
Poor	308	7.4	36	10.9	272	7.1	
Unknown	43	1.0	4	1.2	39	1.0	
*Social network and support*							
Marital status							
Married	3026	72.7	194	59.0	2832	73.8	< *0.001*
Never married/Divorcees	112	2.7	9	2.7	103	2.7	
Widowed	997	23.9	125	38.0	872	22.7	
Unknown	30	0.7	1	0.3	29	0.8	
Living with							
No-one	425	10.3	50	15.4	375	9.9	*0.002*
Others	3705	89.7	275	84.6	3430	90.1	
Frequency of visiting children or other relatives							
Everyday	1134	27.5	96	29.6	1038	27.3	< *0.001*
2–3 per week	619	15.0	52	16.0	567	14.9	
Once a week	650	15.8	38	11.7	612	16.1	
At least monthly	435	10.6	29	9.0	406	10.7	
Seldom	1050	25.5	72	22.2	978	25.8	
Never	234	5.7	37	11.4	197	5.2	
*Co-morbidities*							
Hypertension (BP ≥ 140/90 mmHg or taking antihypertensive drugs)							
No	2128	51.1	145	44.1	1983	51.7	*0.009*
Yes	1882	45.2	170	51.7	1712	44.6	
Unknown	155	3.7	14	4.3	141	3.7	
Heart disease							
No	3524	84.6	279	84.8	3245	84.6	*0.897*
Yes	545	13.1	40	12.2	505	13.2	
Unknown	96	2.3	10	3.0	86	2.2	
Diabetes							
No	3878	93.1	303	92.1	3575	93.2	*0.601*
Yes	228	5.5	20	6.1	208	5.4	
Unknown	59	1.4	6	1.8	53	1.4	
Activity of daily living (ADL) (score)							
0	3713	89.1	241	73.3	3472	90.5	< *0.001*
1–4	295	7.1	38	11.6	257	6.7	
≥ 5	157	3.8	50	15.2	107	2.8	
GMS-AGECAT diagnosis—depression							
Non-depression	3831	92.0	297	90.3	3534	92.1	*0.435*
Depression-subcase	126	3.0	10	3.0	116	3.0	
Depression-case	183	4.4	19	5.8	164	4.3	
Unknown	25	0.6	3	0.9	22	0.6	
GMS-AGECAT diagnosis -Dementia							
Non-dementia	3317	79.6	227	69.0	3090	80.6	< *0.001*
Dementia-subcase	417	10.0	38	11.6	379	9.9	
Dementia-case	406	9.7	61	18.5	345	9.0	
Unknown	25	0.6	3	0.9	22	0.6	
*Dietary variables*							
Meat consumed over the past two years							
Never eat	710	17.2	70	21.5	640	16.8	*0.222*
Once a week or less	1387	33.6	110	33.8	1277	33.6	
> Once a week and < daily	1129	27.3	78	24.0	1051	27.6	
Once a day	631	15.3	48	14.8	583	15.3	
> Daily	274	6.6	19	5.8	255	6.7	
Fish consumed over the past two years							
Never eat	988	23.9	98	30.2	890	23.4	*0.014*
Once a week or less	1327	32.1	110	33.8	1217	32.0	
> Once a week and < daily	1209	29.3	77	23.7	1132	29.7	
Once a day	446	10.8	26	8.0	420	11.0	
> Daily	161	3.9	14	4.3	147	3.9	
Fresh vegetables consumed over the past two years							
Never eat	20	0.5	1	0.3	19	0.5	*0.360*
Once a week or less	86	2.1	11	3.4	75	2.0	
> Once a week and < daily	207	5.0	18	5.6	189	5.0	
Once a day	1685	40.8	122	37.7	1563	41.1	
> Daily	2127	51.6	172	53.1	1955	51.4	
Fruits consumed over the past two years							
Never eat	485	11.8	50	15.4	435	11.5	*0.113*
Once a week or less	1268	30.8	105	32.4	1163	30.6	
> Once a week and < daily	1105	26.8	75	23.1	1030	27.1	
Once a day	969	23.5	68	21.0	901	23.7	
> Daily	292	7.1	26	8.0	266	7.0	

*p values in the Chi-square test are calculated based on available data, not including “Unknown” data

^†^Body mass index (BMI) (categories cut-off point) [42]

^‡^Low level of income defined as those having a poor annual income or a serious financial problem in the last 2 years, while high level included those who were not in the low level of income

Each food category numbers do not sum up to 4165 due to unknown responses

Table 2 shows numbers, mortality rates, and adjusted HRs among the three groups of participants with different levels of fish consumption. There were significant differences in mortality rate among these groups (*p* = 0.011): 34.4 per 1000 person-years in participants who “never eat” fish over the past two years, 28.4 in participants with “a little” fish intake and 20.8 in participants with “a lot” fish intake. Compared to those with “never eat” fish over the past two years, the age-sex adjusted HR of all-cause mortality in participants with “a little” fish consumption was 0.70 (95% CI 0.53–0.93) and in “a lot” 0.56 (0.42–0.74). After further adjustment for socioeconomic status, social support, lifestyles, and BMI, these HRs were slightly increased (Model 2 in Table 2). Adding co-morbidities, meat, vegetables, and fruits consumption for further adjustment (Model 3), the matched HRs were 0.79 (0.53–1.18) and 0.58 (0.34–0.96), respectively. In the model, there were no significant interaction effects of fish consumption with dementia on all-cause mortality.

**Table 2:** Numbers of death and adjusted hazard ratios of mortality in older people with different levels of fish consumption

Fish intake over the past two years	Nos participants (death)	Person-years (mortality*)	HR^1†^ 95% CI	HR^2†^ 95% CI	HR^3†^ 95% CI
Never eat	988 (98)	2848.5 (34.4)	1.00	1.00	1.00
A little^#1^	1327 (110)	3875.8 (28.4)	0.70 0.53–0.93	0.74 0.53–1.04	0.79 0.53–1.18
A lot^#2^	1816 (117)	5633.9 (20.8)	0.56 0.42–0.74	0.59 0.38–0.91	0.58 0.34–0.96
Total	4131 (325)	12,358.3 (26.3)			

*Mortality rate per 1000 person-years

^#1^Including those of “Once a week or less”

^#2^Including those of “ > Once a week and < daily”, “Daily” and “ > Daily”

HR^1^: Adjusted for age (cont.) and sex

HR^2^: Adjusted for age (cont.), sex, province, urban–rural living, educational level, occupational class, income, smoking status, alcohol consumption, BMI, marital status, and frequency of visiting children or other relatives

HR^3^: Adjusted for age (cont.), sex, province, urban–rural living, educational level, occupational class, income, smoking status, alcohol consumption, BMI, marital status, frequency of visiting children or other relatives, hypertension, heart disease, diabetes, activity of daily living, depression (case and subcase), dementia (case and subcase), and consumption of meat, vegetables, and fruits

The findings of a separate data analysis by baseline dementia can be seen in Tables 3 and 4. Table 3 shows numbers, mortality rates, and adjusted HRs among the three groups of non-demented participants with different levels of fish consumption; fully adjusted HR of all-cause mortality was 0.85 (0.55–1.31) in fish consumption of “a little” and 0.57 (0.33–0.98) at “a lot” compared to those that “never eat” over the past two years. Reduced HRs of mortality by increased consumption of fish in participants without dementia were similar to those in all participants (Table 2).

**Table 3:** Numbers of death and adjusted hazard ratios in older people without dementia in China

Fish intake over the past two years	Nos participants (death)	Person-years (mortality*)	HR^1†^ 95% CI	HR^2†^ 95% CI	HR^3†^ 95% CI
Never eat	746 (69)	2176.8 (31.7)	1.00	1.00	1.00
A little^#1^	1046 (74)	3096.6 (23.9)	0.67 0.48–0.93	0.77 0.53–1.13	0.85 0.55–1.31
A lot^#2^	1512 (81)	4774.7 (17.0)	0.49 0.36–0.68	0.55 0.34–0.88	0.57 0.33–0.98
Total	3304 (224)	10,048.1(22.3)			

*Mortality rate per 1000 person-years

^#1^Including those of “once a week or less”

^#2^Including those of “ > once a week and < daily”, “daily” and “ > daily”

HR^1^: Adjusted for age (cont.) and sex

HR^2^: Adjusted for age (cont.), sex, province, urban–rural living, educational level, occupational class, income, smoking status, alcohol consumption, BMI, marital status, and frequency of visiting children or other relatives

HR^3^: Adjusted for age (cont.), sex, province, urban–rural living, educational level, occupational class, income, smoking status, alcohol consumption, BMI, marital status, frequency of visiting children or other relatives, hypertension, heart disease, diabetes, activity of daily living, depression (subcase and cases),  dementia subcase, and consumption of meat, vegetables, and fruits

**Table 4:** Number of death and adjusted hazard ratios in older people with dementia in China

Fish intake over the past 2 years	Nos participants (death)	Person-years (mortality*)	HR^1†^ 95% CI	HR^2†^ 95% CI	HR^3†^ 95% CI
Never eat	123 (17)	327.5 (51.9)	1.00	1.00	1.00
A little^#1^	139 (20)	388.0 (51.4)	0.94 0.41–2.15	0.94 0.34–2.55	1.05 0.30–3.66
A lot^#2^	143 (24)	387.8 (61.9)	0.97 0.45–2.09	1.05 0.30–3.70	1.36 0.28–6.60
Total	405 (61)	1103.3 (55.3)			

*Mortality rate per 1000 person-years

^#1^Including those of “once a week or less”

^#2^Including those of “ > once a week and < daily”, “daily” and “ > daily”

HR^1^: Adjusted for age (cont.) and sex

HR^2^: Adjusted for age (cont.), sex, province, urban–rural living, educational level, occupational class, income, smoking status, alcohol consumption, BMI, marital status, frequency of visiting children, or other relatives

HR^3^: Adjusted for age (cont.), sex, province, urban–rural living, educational level, occupational class, income, smoking status, alcohol consumption, BMI, marital status, frequency of visiting children or other relatives, hypertension, heart disease, diabetes, activity of daily living, depression, and consumption of meat, vegetables, and fruits

However, the data from 405 participants with dementia at baseline showed no association of fish intake with all-cause mortality (Table 4); age-sex HR was 0.94 (0.41–2.15) in those with consumption of fish at “a little” and 0.97 (0.45–2.09) at “a lot” compared to those “never eat”, while with more confounders adjusted for, the association between consumption of fish and all-cause mortality became positive, but not statistically significant, and the fully adjusted HR of all-cause mortality was 1.05 (0.30–3.66) in fish consumption of “a little” and 1.36 (0.28–6.60) at “a lot” compared to those that “never eat” over the past 2 years. There were no significant differences in the HRs between participants with and without dementia; ratio of HRs in the fish consumption of “a little” was 1.24 (95% CI 0.33–4.64), *p* = 0.754, and in “a lot” fish consumption 2.39 (0.45–12.69), *p* = 0.308.

## Discussion

Our community-based cohort study from the five provinces of China revealed that older people with increased fish intake had reduced all-cause mortality. The association is independent of other factors. Stratifying data analysis for the dementia status showed that the impact of fish consumption on survival was more obvious in people who were free of dementia, and there was no association of fish consumption with all-cause mortality in people with dementia.

Previous studies examining the association between consumption of fish and reduced all-cause mortality were mostly undertaken in HICs and in young and middle age groups of populations [8]. Many [8, 9, 26], but not all [27, 28] showed an inverse relationship between fish consumption and all-cause mortality. In a US Chicago Western Electric Study of 1822 male participants aged 40 to 55 years with a follow-up period of 30 years, Daviglus et al. [29] found a non-significant reduction in the risk of all-cause mortality (RR 0.85, 0.64–1.10) when the highest fish consumption was compared with the lowest fish consumption. A non-significant inverse association of fish consumption with all-cause mortality was also found among 17,611 participants aged 32–46 years with 22 years follow-up period in a US National Health and Nutrition Examination Survey (NHANES III), when the highest fish consumption level was compared with the lowest consumption level (HR 0.93, 0.78–1.11) [30]. Other studies including mixed-age groups of population showed more significant association of fish intake with reduced all-cause mortality [31–33]. The US Southern Community Cohort Study (SCCS) followed up 77,604 participants aged 40–79 years for 5.5 years and showed a significant inverse association of total fish consumption with all-cause mortality (adjusted HR 0.92, 0.84–1.00 in the highest quintile of fish consumption versus the lowest quintile) [32]. In a US Vitamins and Lifestyle cohort Study (VITAL Study) of 70,495 participants aged 50–76 years with a follow-up period of 5 years, Bell et al. [31] found a significant reduction in the risk of all-cause mortality (HR 0.86, 0.76–0.98) when the highest fish consumption was compared with the lowest fish consumption. The discrepancies in the findings of the association between fish consumption and all-cause mortality among these studies conducted in HICs could be related to various characteristics within the study population (e.g., age, socioeconomic status), sample size, follow-up duration, confounding adjustment, and data analysis. Furthermore, few of them examined the consumption of fish in older age associated with all-cause mortality, particularly in LMICs. The data of our cohort study in China showed the inverse association between fish consumption in older age and all-cause mortality. The impact of fish intake on reduced all-cause mortality could be from the effects of readily available omega-3 Poly Unsaturated Fatty Acid (PUFA) constituents contained in fish on multiple chronic diseases (such as CVD [1, 34], diabetes [4, 5], respiratory diseases [2], mental illnesss [6], and dementia [7]), resulting from their anti-inflammatory [35], anti-atherosclerotic, antithrombotic [36], and antiarrhythmic and antiatherogenic properties [37, 38] These would help to prevent the development of those chronic diseases and then reduce mortality. The finding of the current study has contributed and filled the gaps in the literature.

On analysing the data of older people with and without dementia separately, we found that the impact of fish intake on reduced all-cause mortality was more obvious in people without dementia at baseline. This may support the pathway of the impact via preventing chronic diseases, including dementia [7]. However, in people with dementia, we have not observed such an inverse association between fish consumption and all-cause mortality, and in contrast found a non-significant association of fish consumption with increased mortality, which could be due to a possible reverse association between dementia severity (or more co-morbidities) and fish consumption or the potential adverse effects of fish intake (e.g., heavy metal contamination). This requires further exploration. As far as we know, no study has been done to examine the impact of fish consumption on all-cause mortality in people with dementia. Previously, a meta-analysis study [39] examined the impact of fish consumption on all-cause mortality among diabetic patients and found a reduced risk of all-cause mortality in the highest category of fish consumption versus the lowest (0.86, 0.76–0.96). A lack of an association between fish consumption and all-cause mortality in older people with dementia may reflect the nature of dementia, i.e., its prognosis would be deteriorating with no effective treatment and intervention. Our data suggest that future research should stratify data analysis according to co-morbidities, particularly dementia, to examine the impact of fish intake on all-cause mortality in populations.

### Strengths and limitations of the study

The main contribution of this study is to identify the impact of increased consumption of fish on all-cause mortality in older people from LMICs, particularly including rural areas in China. To the best of our knowledge, it is the first study in the world to examine the association of fish consumption with all-cause mortality in people with dementia. Our study included many important confounders for adjustment and the findings would be robust. Our study has some limitations. First, in the baseline health survey, participants’ self-reported frequencies of dietary information on the consumption of fish were used for analysis. This may have caused a misclassification of the level of fish intake, which would make our findings of the association to tend towards the null hypothesis. Second, our cohort study did not collect baseline data for types of fish (e.g., lean, fatty-fish, and seafood), quantity of fish, and the omega-3 supplements consumed, like some other studies [40, 41]. Thus, we cannot infer which types and quantity of fish were associated with all-cause mortality. Future research is required to assess which types of fish intake in older age would be significantly associated with all-cause mortality in people with and without dementia. Third, due to the absence of the total energy intake in the data collection, we could not adjust for it and its residual effect could not be excluded from the association between fish consumption and all-cause mortality. However, we adjusted for the consumption of meat and vegetable/fruit and the residual effect would be minimised. Future research is needed to include the total energy intake for adjustment to confirm the association of fish consumption with all-cause mortality in older people and in people with dementia.

### Conclusion

This study has demonstrated an inverse association of fish intake at older age with all-cause mortality in older people, but not in people with dementia. The findings suggest that it would be better to increase the consumption of fish in older people in general, prior to the development of dementia in the hope of preventing dementia and prolonging life.
